# Microsecond Molecular Dynamics Simulations of Mg^2+^- and K^+^- Bound E1 Intermediate States of the Calcium Pump

**DOI:** 10.1371/journal.pone.0095979

**Published:** 2014-04-23

**Authors:** L. Michel Espinoza-Fonseca, Joseph M. Autry, David D. Thomas

**Affiliations:** Department of Biochemistry, Molecular Biology, and Biophysics, University of Minnesota, Minneapolis, Minnesota, United States of America; Instituto de Tecnologica Química e Biológica, UNL, Portugal

## Abstract

We have performed microsecond molecular dynamics (MD) simulations to characterize the structural dynamics of cation-bound E1 intermediate states of the calcium pump (sarcoendoplasmic reticulum Ca^2+^-ATPase, SERCA) in atomic detail, including a lipid bilayer with aqueous solution on both sides. X-ray crystallography with 40 mM Mg^2+^ in the absence of Ca^2+^ has shown that SERCA adopts an E1 structure with transmembrane Ca^2+^-binding sites I and II exposed to the cytosol, stabilized by a single Mg^2+^ bound to a hybrid binding site I′. This Mg^2+^-bound E1 intermediate state, designated E1**•**Mg^2+^, is proposed to constitute a functional SERCA intermediate that catalyzes the transition from E2 to E1**•**2Ca^2+^ by facilitating H^+^/Ca^2+^ exchange. To test this hypothesis, we performed two independent MD simulations based on the E1**•**Mg^2+^ crystal structure, starting in the presence or absence of initially-bound Mg^2+^. Both simulations were performed for 1 µs in a solution containing 100 mM K^+^ and 5 mM Mg^2+^ in the absence of Ca^2+^, mimicking muscle cytosol during relaxation. In the presence of initially-bound Mg^2+^, SERCA site I′ maintained Mg^2+^ binding during the entire MD trajectory, and the cytosolic headpiece maintained a semi-open structure. In the absence of initially-bound Mg^2+^, two K^+^ ions rapidly bound to sites I and I′ and stayed loosely bound during most of the simulation, while the cytosolic headpiece shifted gradually to a more open structure. Thus MD simulations predict that both E1**•**Mg^2+^ and E**•**2K^+^ intermediate states of SERCA are populated in solution in the absence of Ca^2+^, with the more open 2K^+^-bound state being more abundant at physiological ion concentrations. We propose that the E1**•**2K^+^ state acts as a functional intermediate that facilitates the E2 to E1**•**2Ca^2+^ transition through two mechanisms: by pre-organizing transport sites for Ca^2+^ binding, and by partially opening the cytosolic headpiece prior to Ca^2+^ activation of nucleotide binding.

## Introduction

**Figure 1:**
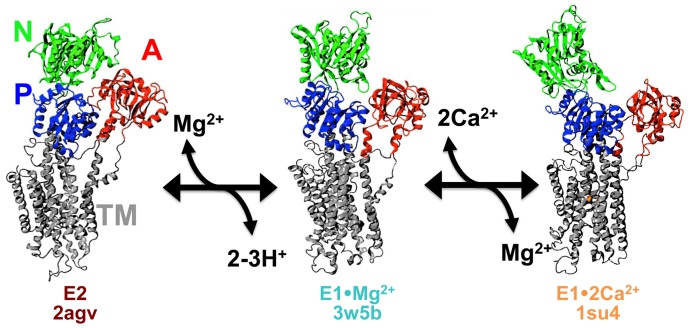
Proposed structural model for Mg^2+^ facilitation of the E2-to-E1•2Ca^2+^ transition. During the transition from Ca^2+^-free E2 (left) to Ca^2+^-bound E1 (right), a Mg^2+^ bound intermediate is proposed to neutralize negative charges resulting from deprotonation of acidic residues in the Ca^2+^ -binding sites. This Mg^2+^-bound E1 state, designated E1·Mg^2+^ (center), features an open cytosolic-facing water-access channel to the cation-binding sites and a partially open headpiece conformation (N, P, and A domains). In the presence of cytosolic Ca^2+^, Mg^2+^ is exchanged for two Ca^2+^, facilitating formation of E1·2Ca^2+^. SERCA is colored according to its four functional domains: N (green), P (blue), A (red), and TM (grey). The PDB accession code for each structural intermediate is indicated. Adapted from [15].

P-type ATPases are responsible for active transport of a specific ion, such as Ca^2+^, Na^+^, or K^+^, against its concentration gradient [1], [2]. The prototype of this family is the sarcoendoplasmic reticulum Ca^2+^-ATPase (SERCA), the calcium pump that is responsible for the active and selective transport of Ca^2+^ from the cytosol into the sarcoplasmic reticulum of muscle cells, or into the endoplasmic reticulum of non-muscle cells [3]. Structurally, SERCA contains four functional domains: nucleotide-binding (N), phosphorylation (P), actuator (A), and transmembrane (TM) (**Figure. 1**) [4]. SERCA binds two Ca^2+^ ions in the TM domain, which are pumped into the SR lumen using energy derived from hydrolysis of one ATP molecule and the counter-transport of 2–4 protons [5], [6]. The catalytic cycle of SERCA involves a major structural transition between two key conformations: low Ca^2+^ affinity E2, with binding sites exposed to the lumen, and high Ca^2+^ affinity E1, with binding sites exposed to the cytosol. This E2→E1 transition is driven by Ca^2+^/H^+^ exchange and may include steps facilitated by other cations [7], [8], [9].

Experimental and computational studies have provided evidence that structural changes necessary for coupling of Ca^2+^ binding to ATP hydrolysis are linked to structural dynamics of the cytosolic headpiece [2]. In the proposed catalytic cycle, the transition between the E2 ground state and the Ca^2+^-activated E1•2Ca^2+^ state includes an apo E1 intermediate. The negatively charged Ca^2+^ binding sites of SERCA probably need to be neutralized for formation of apo E1, but the occupancy of TM binding sites remains unclear for apo E1 (H^+^, Mg^2+^, K^+^, and/or Na^+^) [7], [9], [10], [11]. Given the difficulty in obtaining crystal structures of apo E1, several groups have performed atomistic computer simulations to study the structural dynamics of this intermediate, starting from the crystal structure of E1•2Ca^2+^ but removing Ca^2+^ [12], [13]. These studies provided key predictions on Ca^2+^ binding and allosteric coupling of domain dynamics, but the time scales used in the atomistic simulations were too short for Ca^2+^-free E1 to populate a fully relaxed E1 intermediate state [12], [13]. Coarse-grained simulations were used to simulate the transition path between E2 and E1, but that study did not take into consideration important atomistic factors, such as changes in protonation states of the Ca^2+^-binding sites and the explicit inclusion of metal ions [14].

Super-physiological concentrations of Mg^2+^ have been used recently to obtain crystal structures of SERCA in proposed apo E1 conformations, with high-affinity Ca^2+^ binding sites exposed to the cytosol without bound Ca^2+^ [15], [16]. One crystal structure, obtained in the presence of 40 mM Mg^2+^, shows an apo E1 structure stabilized with a single bound Mg^2+^ [15]. Another crystal structure, obtained in 75 mM Mg^2+^, shows an apo E1 structure stabilized by two bound Mg^2+^ [16]. The ionized Mg^2+^ concentration in skeletal muscle cytosol is ∼1–2 mM [17], [18]. Electrode-based measurements of cation binding by SERCA and its mutants indicate that only one Mg^2+^ ion binds to the TM Ca^2+^ binding sites in solution [19]. The crystal structure of the E1 intermediate state with one bound Mg^2+^, designated E1•Mg^2+^, features a hybrid cation-binding site I′ occupied by a single Mg^2+^, and a semi-open cytosolic headpiece conformation that is not suitable for ATP utilization [15] (**Figure. 1**). Thus it was proposed that, following the pH-dependent E2-to-E1(apo) transition of SERCA, Mg^2+^ binding to the Ca^2+^-binding site I′ is required to stabilize the apo E1 intermediate state in the absence of Ca^2+^ (**Figure. 1**) [15]. However, the E1•Mg^2+^ crystal was obtained in the presence of much higher Mg^2+^ concentration than found in muscle cytosol, and in the absence of Ca^2+^ and K^+^, so the functional significance of this structure remains unclear, particularly since high concentrations of Mg^2+^ have been reported to inhibit SERCA [6], [20], [21]. On the other hand, K^+^ binding to Ca^2+^ transport site(s) is reported to activate SERCA [7], [22], although K^+^ binding to TM sites has not been detected by x-ray crystallography. Thus, major questions remain regarding the role of Mg^2+^ and K^+^ in H^+^/Ca^2+^ exchange: Are the E1•Mg^2+^ and E1•K^+^ intermediate states populated in solution? If so, what mechanistic role(s) do they play in transport? To address these questions, we have performed two all-atom MD simulations of the E1•Mg^2+^ SERCA crystal structure, starting in the presence or absence of initially-bound Mg^2+^, in a solution containing physiologically appropriate concentrations of other ions (100 mM K^+^, 5 mM Mg^2+^, and 110 mM Cl^−^).

## Methods

### Construction of the E1•Mg^2+^ system

We used the crystal structure of recombinant E1•Mg^2+^ (i.e., free of sarcolipin (SLN) [15]; PDB code: 3w5b) to simulate the dynamics of E1 in the presence of a single Mg^2+^ ion bound to site I′. Although the structures of recombinant E1•Mg^2+^ and SLN-bound E1•Mg^2+^ are very similar, the A domain is slightly rotated to populate an orientation between E1•Mg^2+^ and E1•2Ca^2+^ [15]. However, preliminary rounds of short MD simulations showed that this difference in A domain orientation between native and recombinant E1•Mg^2+^ is small (data not shown), indicating that the crystal structure of recombinant E1•Mg^2+^ is an adequate starting structure to simulate the dynamics of E1. To determine the effect of metal ion binding on the structural dynamics of E1, we removed the ATP analog trinitrophenyl adenosine monophosphate (TNP-AMP) and the Mg^2+^ ion bound to the phosphate group of TNP-AMP. We also removed two crystallographic water molecules located in the first coordination shell of the remaining Mg^2+^, because water-Mg^2+^ interatomic distances did not converge after exhaustive energy minimization rounds. We used PROPKA to adjust the protonation states of ionizable residues, corresponding to pH 7.5 [23], [24]. Ca^2+^-binding acidic residues E771, D800, and E309 were kept unprotonated, whereas residue E908 was modeled in its protonated form. Mg^2+^-bound SERCA inserted in a pre-equilibrated POPC bilayer composed of 376 lipid molecules; protein-lipid systems were solvated using ∼50,000 TIP3P water molecules. K^+^, Mg^2+^, and Cl^−^ ions were added to produce concentrations of 100 mM, 5 mM, and 110 mM, respectively. CHARMM36 force field topologies and parameters were used for the protein [25], lipid [26], water, K^+^ and Cl^−^. In addition, we used a set of new CHARMM parameters for Mg^2+^ developed by Allnér et al. [27] This new set of parameters for Mg^2+^ aimed at correcting the Mg^2+^-water exchange rate, as previous parameters do not correctly capture the water exchange kinetics between the first coordination shell and bulk water [27].

### Construction of the apo E1 system

We used the crystal structure of E1•Mg^2+^ (PDB code: 3w5b) to construct a three-dimensional model of the E1 intermediate state in the absence of bound Mg^2+^. To simulate this state, we removed the Mg^2+^ ions located in the phosphorylation site and the TM binding site I′. In addition, the ATP analog trinitrophenyl adenosine monophosphate was removed from the crystal structure. Ca^2+^-binding acidic residues E771, D800 and E309 were kept unprotonated, whereas residue E908 was modeled in its protonated form. This structure of SERCA was inserted in a POPC bilayer and solvated using ∼50,000 TIP3P water molecules. K^+^, Mg^2+^, and Cl^−^ ions were added to produce concentrations of 100 mM, 5 mM, and 110 mM, respectively. To prevent structural artifacts associated with the charge imbalance produced by Mg^2+^ removal from the Ca^2+^-binding sites, we performed a 5 ns equilibration cycle of the system with the protein heavy atoms harmonically restrained using a force constant of 2000 kcal mol^−1^ nm^−2^. This short equilibration cycle resulted in the binding of a single K^+^ ion to site I′ of SERCA and virtually no changes in the atomic positions of each residue of the protein. Therefore, SERCA with a single bound K^+^ ion was used as a starting model for the simulation of apo E1 in the absence of bound Mg^2+^.

### Molecular dynamics simulations

We performed MD simulations by using the program NAMD 2.9 [28]. We used periodic boundary conditions [29], particle mesh Ewald [30], [31], a nonbonded cutoff of 1 nm, and a 2 fs time step. A temperature of 310K was maintained with a Langevin thermostat, and a constant pressure of 1 atm was controlled with an anisotropic Langevin piston barostat. The systems were first subjected to energy minimization for 2000 steps, followed by gradually warming up of the systems to a target temperature of 310K. This procedure was followed by several cycles of equilibration with the protein heavy atoms harmonically restrained using force constants of 1000, 500, 20, 5 and 0 kcal mol^−1^ nm^−2^, respectively; each equilibration cycle was performed for 0.01 µs. Unrestrained production runs for E1•Mg^2+^ and apo E1 were performed for 1 µs.

## Results

### Mg^2+^ and K^+^ interactions with SERCA in the Ca^2+^-binding sites

**Figure 2:**
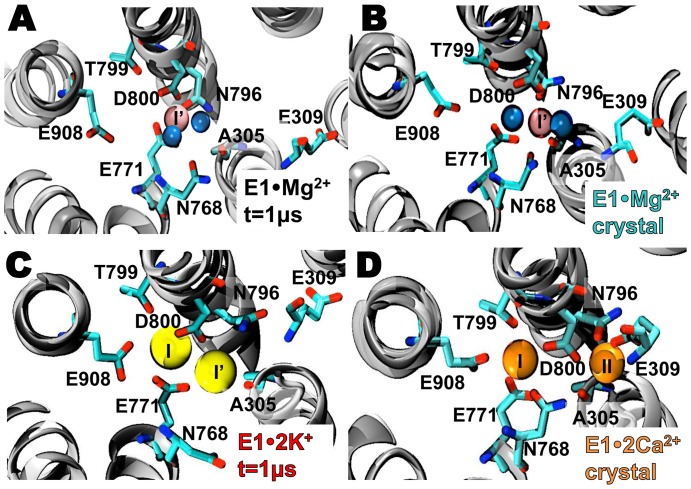
Mg^2+^ and K^+^ interactions with SERCA in the Ca^2+^-binding sites. (A) E1·Mg^2+^ structure with Mg^2+^ bound to hybrid binding site I′ (A) at the end of the MD simulation and (B) in the crystal structure 3w5b [15]. Mg^2+^ ions and coordinating water oxygen atoms are shown as *pink* and *blue spheres*, respectively. (C) Structure of apo E1 at the end of the MD simulation with two bound K^+^ ions, K^+^_(I)_ and K^+^_(I′)_, shown as *yellow spheres*. (D) Crystal structure of E1·2Ca^2+^ (1su4) showing the location of the Ca^2+^-binding sites I and II, with Ca^2+^ ions shown as *orange spheres* [40]. In all panels, the TM helices are represented by *grey ribbons* and cation-binding residues are shown as *sticks*.

We investigated cation interactions with the Ca^2+^-binding sites of E1•Mg^2+^ under solution conditions approximating the cytosol during muscle relaxation (100 mM K^+^, 5 mM Mg^2+^, 110 mM Cl^−^, absence of Ca^2+^). Starting from the Mg^2+^-bound crystal structure, we found that the Mg^2+^ ion remained bound to hybrid site I′ during the entire 1 µs simulation (**Figure 2A**). The Mg^2+^ ion showed a restricted mobility in site I′, with an average root-mean square fluctuation (RMSF) value of 0.04 nm. MD simulation of E1•Mg^2+^ demonstrated that Ca^2+^-binding site II remains cation-free during the entire simulation (**Figure 2A and B**), indicating that a single bound Mg^2+^ is sufficient to stabilize SERCA in a Ca-free apo E1 state. The average position of Mg^2+^ in the MD simulation is similar to that of the crystal structure, with a root mean square deviation (RMSD) difference ≤0.1 nm between the crystal structure and the MD trajectory (**Figure 2**, **Table 1**). The estimated average interaction energy (*E*_int_) of Mg^2+^ in site I′ is −950 kcal mol^−1^, while the average *E*_int_ of Ca^2+^ bound to the Ca^2+^-binding site I is -830 kcal mol^−1^ [13], suggesting that Mg^2+^ has a slow off rate from site 1′ and thus E1•Mg^2+^ represents an inhibitory, not activating, E1 intermediate.

**Table 1:** Interatomic distances between metal ions and coordinating oxygen atoms of SERCA.

	Metal ion					
Residue (oxygen type)	Mg^2+^ (MD^1^)	Mg^2+^ crystal^2^	K^+^_(I)_ (MD^3^)	K^+^_(I′)_ (MD^3^)	Ca^2+^_(I)_ crystal^4^	Ca^2+^_(II)_ crystal^4^
**A305 (O_Backbone_)**	-	0.26	-5	0.26±0.09	-	-
**N768 (O_δ1_)**	-	0.23	-	-	0.25	-
**E771 (O_ε1_)**	0.20±0.01	0.25	0.27±0.02	-	-	-
**E771 (O_ε2_)**	0.20±0.02	-	-	-	0.24	-
**N796 (O_δ1_)**	0.21±0.01	0.26	-	-	-	0.24
**T799 (O_γ_)**	-	-	0.29±0.06	-	0.24	-
**D800 (O_δ1_)**	0.20±0.01	-	0.30±0.10	0.30±0.06	0.23	-
**D800 (O_δ2_)**	0.20±0.01	-	0.30±0.09	-	-	0.23
**E908 (O_ε1_)**	-	-	0.29±0.06	-	-	-

Distances were calculated for MD simulations and crystal structures. Non-bonding distances were removed from Table (≥0.21 nm for Mg^2+^, ≥0.30 nm for K^+^, and ≥0.25 nm for Ca^2+^).

1 MD simulation of E1•Mg^2+^ structure. Errors are ± SD.

2 X-ray crystal structure of E1•Mg^2+^ (3w5b).

3 MD simulation of apo E1 structure. Errors are ± SD.

4 X-ray crystal structure of E1•2Ca^2+^ (1su4).

5 Non-bonding distances are not shown in Table.

The bound Mg^2+^ ion has octahedral coordination geometry and interacts with six coordinating oxygen atoms for most of the simulation time. The six coordinating ligands for Mg^2+^ are three carboxylic oxygen atoms from residues E771 and D800, the carbonylic oxygen from residue N796, and two water molecules (**Figure 2A**). In our simulation, a nanosecond time scale rotation of the carboxylic group of D800 was observed about the C_β_-C_γ_ bond, which allows both Oδ_1_ and Oδ_2_ to switch positions in the first coordination shell of Mg^2+^. Nevertheless, the Oδ_1_−Oδ_2_−Oδ_1_ switching does not affect the coordination geometry or the mobility of Mg^2+^ bound to site I′. The average SERCA-Mg^2+^ distances in the MD trajectory (**Table 1**) are in excellent agreement with metal-donor atom target distances expected for carboxylic oxygen-Mg^2+^ (0.21 nm) and carbonylic oxygen-Mg^2+^ (0.23 nm) in proteins [32]. Two differences in the first coordination shell of Mg^2+^ were observed between the crystal structure and MD simulation of E1•Mg^2+^. First, in the crystal structure, the backbone oxygen of A305 and the side chain of N768 belong to the coordination shell of Mg^2+^ (**Figure 2B**). Second, in the course of the MD simulation, A305 and N768 are replaced by two water molecules in the first coordination shell of Mg^2+^ (**Figure 2A**). These rearrangements of coordinating residues and waters around the bound Mg^2+^ ion are not surprising because the MD simulation is run in solution, and because the 0.32 nm resolution of the crystal structure contains uncertainty in the precise coordination shell of Mg^2+^ [15].

In the absence of initially-bound Mg^2+^,we found that two potassium ions, K^+^_(I)_ and K^+^_(I′)_, bind in novel fashion to unique rearrangements of the two Ca^2+^-binding sites (**Figure 2C**). Binding of two K^+^ ions is probably required to mimic charge neutralization produced by bivalent metal ions in the calcium sites. We designate this K^+^-bound intermediate as E1•2K^+^. We found that K^+^_(I)_ and K^+^_(I′)_ binding to the TM sites follow TM1 pathway [33], where K^+^ ions are guided by E55, E58, E59 and E109 toward site II before reaching sites I and I′, respectively. We did not find any evidence of another entry site to the TM domains (i.e., via TM8-9 [34]). K^+^_(I)_ interacts with residues E771, T799, D800 and E908 in a location that virtually overlaps with the site occupied by Ca^2+^_(I)_ in E1•2Ca^2+^. (**Figure 2D**). However, the average interaction energy *E*_int_ between K^+^_(I)_ and the Ca^2+^-binding site I is −350 kcal mol^−1^, which is much weaker compared to a *E*_int_ value of −830 kcal mol^−1^ calculated for Ca^2+^ in the same site using a 0.5-µs trajectory of E1•2Ca^2+^ reported previously [13]. K^+^_(I′)_ binds to site I′ at *t* = 0.07 µs; it interacts weakly (*E*_int_ = −320 kcal mol^−1^) with the backbone oxygen of A305, and with A305 and D800 (**Figure 2C** **and** **Table 1**). We found that K^+^_(I′)_ binds in a location 0.3 nm away from the site where a second high-affinity Ca^2+^, Ca^2+^_(II)_, binds in E1•2Ca^2+^ (**Figure 2C and D**). Despite the proximity to this site, we found that K^+^_(I′)_ does not engage residues E309 and N796 in metal ion-SERCA interactions, which is a requirement for metal ion occlusion in the Ca^2+^ -binding site II [35], This indicates that under physiological conditions, K^+^_(I′)_ binding does not induce the formation of the Ca^2+^-binding site II. We did not observe K^+^-Mg^2+^ exchange in either 1 µs MD simulation; however, it is possible that ion exchange at the Ca^2+^-binding sites of SERCA occurs under physiological conditions but in much longer time scales (i.e. hundreds of microseconds to milliseconds).

### Structural dynamics of acidic residues in the Ca^2+^ binding sites of E1•Mg^2+^ and E1•2K^+^

**Figure 3:**
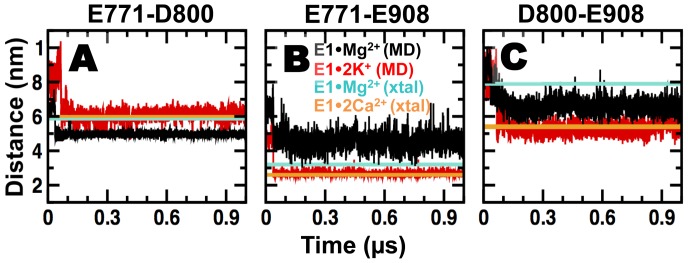
Time-dependent distance evolution of carboxyl-carboxyl pairs between residues E771, D800, and E908. Distance between residues (A) E771-D800 on TM5-TM6, (B) E771-E908 on TM5-TM8, and (C) D800-E908 on TM6-TM8 were calculated for MD simulations E1·Mg^2+^ (black) and E1·K^+^ (red) and compared to crystal structures E1·Mg^2+^ (3w5b in cyan) and E1·2Ca^2+^(average of 1su4 and 1vfp in orange).

The cation binding sites of SERCA are formed by four helices (TM4, TM5, TM6, TM8), each of which contribute a carboxylate side chain. To analyze the effect of Mg^2+^ and K^+^ binding on the structural dynamics of acidic residues that play a central role in Ca^2+^ binding [36], [37], [38], [39], we plotted time-dependent distance evolution of the carboxyl-carboxyl pairs between centrally-positioned residues E771 (TM5), D800 (TM6), and E908 (TM8) (**Figure 3**). E309 (TM4) was analyzed separately, due to its role as “capping” residue of the cytosolic gate. Distances between E771 and D800, were calculated using atoms C_δ_ and C_γ_, respectively. The distance between E771 and E908 (E771-E908) was calculated between the protonated oxygen (O_ε2_) from the carboxylic group of E908 and the atom O_ε1_ from E771. Finally, the distance E800-E908 was calculated between atoms O_ε_ and C_γ_ of E908 and D800, respectively. These distances were chosen based on the spatial arrangement between E771, D800 and E908 in the crystal structure of E1•2Ca^2+^. All inter-residue distances converged in both MD simulations of E1•Mg^2+^ and E1•2K^+^ (**Figure 3**), indicating that the structures shown in **Figure 2A and C** represent equilibrium geometries in solution.

**Figure 4:**
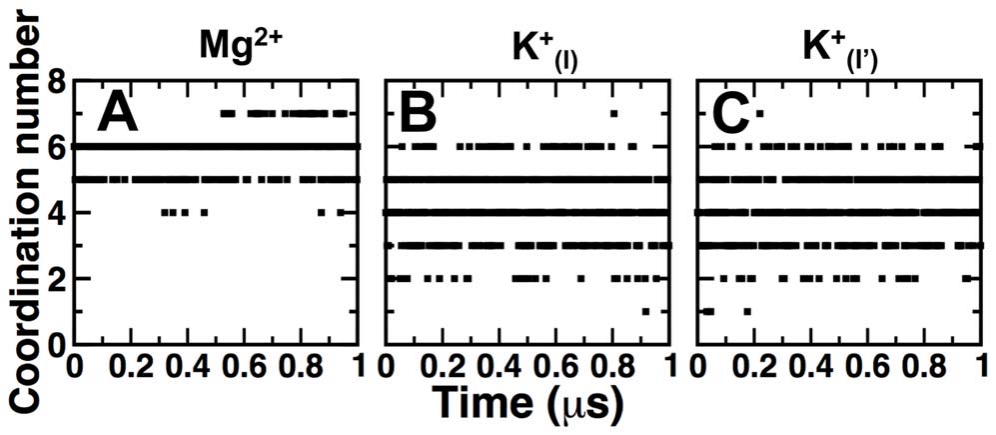
Time dependence of the coordination number for metal ions bound in E1•Mg^2+^ and E1•2K^+^. The coordination number of bound cations was calculated every 0.01 µs during MD simulation using an oxygen-metal cutoff distance of 0.21 nm and 0.30 nm for Mg^2+^ and K^+^, respectively. (A) Mg^2+^ in E1·Mg^2+^. (B, C) K^+^_(I)_ and K^+^_(I′)_ in E1·2K^+^.

The distance between residues D800 and E908 is very similar in E1•Mg^2+^ and E1•2K^+^, with a value of ∼0.65 nm (**Figure 3C**). This value is close to an average distance of 0.67 nm calculated from the crystal structures of E1•2Ca^2+^ [40], indicating that the spatial arrangement between residues D800 and E908 does not depend on the kind of metal ion bound to Ca^2+^-binding site I. However, inter-residue distances of E771-D800 and E771-E908 are different between E1•Mg^2+^ and E1•2K^+^: Mg^2+^ binding shortens the distance between residues E771 and D800 by 0.1 nm (**Figure 3A**), whereas K^+^ binding shortens the distance between E771 and E908 by 0.2 nm (**Figure 3B**). These differences indicate that the spatial arrangement of residues E771-D800 and E771-E908 is sensitive to different metal ions. Further comparison of distances calculated from the MD trajectories with those calculated from the crystal structure of E1•2Ca^2+^ showed that K^+^, but not Mg^2+^, produces a spatial separation between E771, D800, and E908 similar to that induced by Ca^2+^-binding. The differences in spatial separation probably relate to the chemical properties of K^+^ and Mg^2+^. The ionic radius of K^+^ (0.15 nm) is 1.7 and 1.4 times larger than that of Mg^2+^ (0.09 nm) and Ca^2+^ (0.11 nm), respectively [41]. In order to accommodate K^+^_(I)_, Mg^2+^ and Ca^2+^ between acidic residues E771 and D800, it is expected that the E771-D800 distance trend follows E1•2K^+^>E1•2Ca^2+^>E1•Mg^2+^, in agreement with our MD simulations (**Figure 3A**). In addition, we calculated the time series of the coordination numbers for Mg^2+^, K^+^_(I)_ and K^+^_(I′)_ in the Ca^2+^ sites. Coordination numbers were calculated by counting the number of nearest neighbor oxygen atoms surrounding the metal ions using a cutoff distance of 0.21 nm and 0.30 nm for Mg^2+^ and K^+^, respectively. We found that the coordination number of Mg^2+^ in the Ca^2+^ site I′ is fairly constant during the entire simulation time, with coordination numbers of either 5 or 6 (**Figure 4A**). Conversely, we observed a large variability in the coordination numbers of K^+^_(I)_ and K^+^_(I′)_, with values ranging from 2 to 6 (**Figure 4B and C**). Analysis of the percentage of time Mg^2+^, K^+^_(I)_ and K^+^_(I′)_ have coordination numbers between 2 and 6 showed that the K^+^ does not have a strong preference for a specific coordination number even in a structurally restrained environment such as the Ca^2+^ sites (**Table 2**). However, we found that during 80% of the time Mg^2+^ has a coordination number of 6 (**Table 2**). Most common coordination numbers range from 4 to 8 for K^+^ [42] and 6 to 9 for Ca^2+^ [43], [44], but for Mg^2+^ octahedral six-coordination is found to be most prevalent [43], [44], [45], [46], [47], in agreement with our results. Therefore, the large variability in the coordination number of K^+^ and the range overlap with the coordination numbers of Ca^2+^ results in the ability of K^+^_(I)_ to induce local structural changes and interact with site I in a similar fashion as Ca^2+^ does (**Figure 2** and **Figure 3**). On the other hand, constant coordination number and slow oxygen-metal exchange [42] in the coordination shell of Mg^2+^ prevent sites I and I′ from adopting a Ca^2+^-bound-like geometry (**Figure 2** and **Figure 3**). We propose that the combination of these factors allows the Ca^2+^-binding site I to adopt a Ca^2+^-bound-like geometry in the presence of K^+^, but not Mg^2+^.

**Table 2:** Population distribution of the coordination number for metal ions bound in E1•Mg^2+^ and E1•2K^+^ simulation.

	% of time		
Coordination number	Mg^2+^	K^+^_(I)_	K^+^_(I′)_
**2**	0	5	5
**3**	0	20	22
**4**	<1	36	38
**5**	14	29	26
**6**	80	9	8.5
**7**	5	<1	1

**Figure 5:**
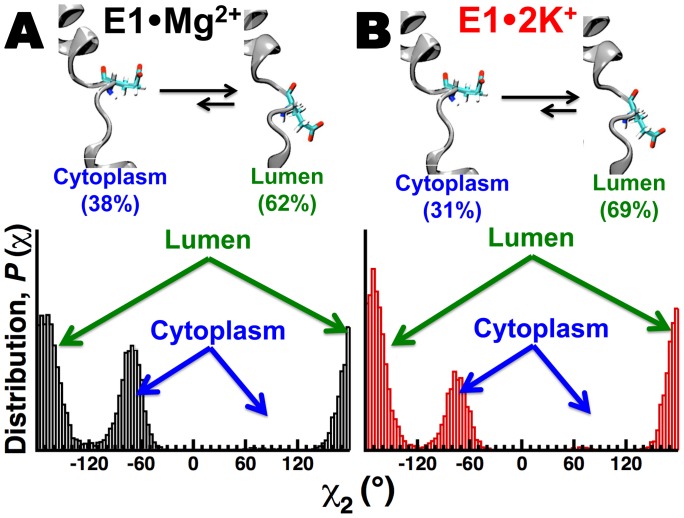
Side-chain dynamics of residue E309. Orientation (top) and population distribution of the dihedral angle *χ*_2_ (bottom) of E309 in the MD simulation of E1·Mg^2+^ (A) and E1·2K.(B). The cartoon on top of each dihedral angle distribution represents the two possible orientations of the carboxylic side chain of E309: toward the cytosol and towards the lumen. The percentage of time spent in each conformation is shown in *parentheses*.

Residue E309 plays a central role in occluding the second Ca^2+^ ion to the Ca^2+^-binding site II [35]. However, we did not observe cation binding to Ca^2+^-binding site II in our simulations. Therefore, we calculated the side chain dihedral angle χ_2_ (defined by atoms C_α_, C_β_, C_γ_ and C_δ_) to evaluate the side-chain dynamics of E309 in the trajectories of E1•Mg^2+^ and E1•2K^+^. χ_2_ angle distributions show that in both E1•Mg^2+^ and E1•2K^+^, the side chain of E309 is in a dynamic equilibrium between two orientations (**Figure 5**): a conformation with the carboxylic group pointing toward the lumen (−180°≤χ_2_≤−120° or +120°≤χ_2_≤+180°), and a side chain orientation where the carboxylic group points toward the cytosol (−119°≤χ_2_≤−40° or +40°≤χ_2_≤+119°). Calculation of percentage of time spent in each orientation showed that E309 spends 62% and 69% of the time facing the lumen in the trajectories of E1•Mg^2+^ and E1•2K^+^, respectively, indicating that the preferred geometry of E309 is the one with the carboxylic group pointing toward the lumenal face of the lipid bilayer. This finding is in agreement with crystal structures showing that E309 points toward the lumenal side of the sarcoplasmic reticulum in the presence of a bound Ca^2+^ ion in site II [15], [16], [48]. Previous MD simulations of SERCA showed that E309 is locked exclusively towards the lumen when Ca^2+^-binding site II is occupied by Ca^2+^ [13], [49], indicating that the freedom of E309 to sample both orientations results from the inability of E1•Mg^2+^ and E1•2K^+^ to lock the E309 side chain in place.

### Structural dynamics of SERCA domains in E1•Mg^2+^ and E1•2K^+^

**Figure 6:**
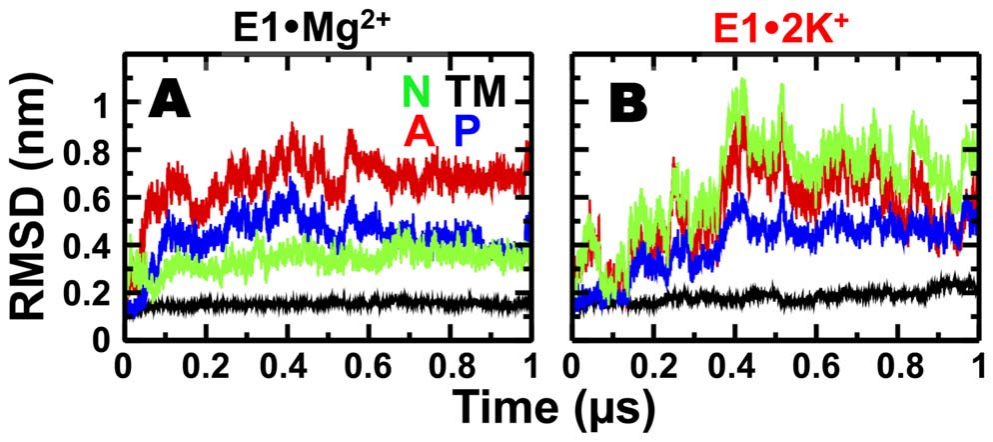
Time-dependent distance evolution of SERCA domains in E1•Mg^2+^and E1•K^+^. RMSD was calculated through simulation trajectories using backbone alignment for TM helices and rigid body domain alignment for cytosolic domains N, A, and, P. Domains are color-coded as indicated in Figure. 1.

To determine the time-dependent structural dynamics of the E1 intermediate with bound Mg^2+^ or K^+^, we calculated the backbone root-mean-square deviations (RMSD) for each functional domain of SERCA in the 1 µs MD simulation trajectories (**Figure 6**). At the beginning of the simulation, the structure of the 10-helix TM domain of E1•Mg^2+^ undergoes a 0.15-nm drift in the picosecond time scale (**Figure 6A**). This modest change in RMSD is attributed to the relaxation of the TM domain in a lipid-water environment. Following this rapid relaxation period, the RMSD values remained virtually unchanged, demonstrating that the transmembrane domain of E1•Mg^2+^ is stable in solution. This result indicates that SLN binding is not necessary to stabilize E1•Mg^2+^, as was recently proposed [15] [16]. The TM domain of E1•2K^+^ also equilibrates in the picosecond time scale; however, the RMSD shifts ≤0.05 nm at different points in the trajectory, indicating that the TM domain has some flexibility in the microsecond time scale. (**Figure 6B**). Nevertheless, the changes in RMSD are the maximum deviation from the crystal structure are relatively small (i.e., RMSD ≤0.25 nm) indicating that K^+^ binding to SERCA does not disrupt the structural integrity of the TM domain (**Figure 6B**).

We observed a large variability in the RMSD values for each domain in the cytosolic headpiece of E1•Mg^2+^ and E1•2K^+^ (**Figure 6**). The N domain of E1•Mg^2+^ undergoes a small spatial rearrangement during the first 0.1 µs of simulation, deviating only ∼0.3 nm from the crystal structure (**Figure 6A**). This initial change in the RMSD is attributed to the relaxation of the N domain in solution. Following this relaxation period (0–0.1 µs), RMSD values remained unchanged in the trajectory, indicating that the position of the N domain is restricted in E1•Mg^2+^, in agreement with relatively low crystallographic B-factors estimated for this domain. We observed large shifts in the RMSD values of A and P domains in the 0.6 µs of the E1•Mg^2+^ trajectory (**Figure 6A**). However, after 0.6 µs the RMSD values of A and P domains settle a plateau around 0.7 and 0.4 nm, respectively, indicating that Mg^2+^ binding also imposes some restrains on the conformational dynamics of A and P in the submicrosecond time scale. The 0.7- and 0.4-nm change in the RMSD of the P and A domains suggests that the relative orientation of the two domains drifts away from the crystal structure orientation.

Analysis of the time-dependent changes in the RMSD E1•2K^+^ showed that the RMSD values of P domain increase to an average plateau value of 0.5 nm during time interval between 0 and 0.4 µs (**Figure 6B**). Conversely, we observed large fluctuations and the absence of a plateau in the RMSD of N and A domains in the E1•2K^+^ (**Figure 6B**). This observations indicate that (a) the relative orientation of N, A and P domains in E1•2K^+^ is very different compared to the crystal structure of E1•Mg^2+^ and (b) in solution, the A and N domains undergo large conformational changes in the sub-microsecond time scale.

### Spatial arrangement of the cytosolic headpiece of E1•Mg^2+^ and E1•2K^+^

**Figure 7:**
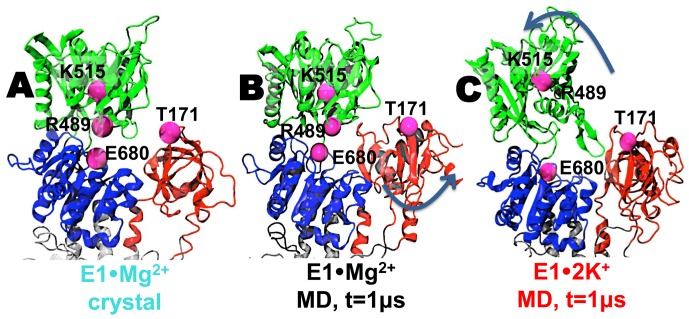
Structural arrangement of the headpiece of E1•Mg^2+^ and E1•2K^+^. (A) E1·Mg^2+^ crystal structure (PDB code: 3w5b). (B) E1·Mg^2+^ at the end of the 1 µs MD simulation; the *blue arrow* shows the direction of the 25° axial rotation of the A domain. (C) E1·2K^+^ at the end of the 1 µs MD simulation; the *blue arrow* shows the direction of the N domain translation that increases ATP binding site accessibility. The *magenta spheres* indicate the position of residues used to calculate interdomain distance distributions shown in **Figure 8**. N, A and P domains are colored in green, red and blue, respectively.

Analysis of the RMSD evolution revealed that binding of Mg^2+^ or K^+^ to the Ca^2+^-binding sites are capable of maintaining the structural integrity of the TM domain of E1 SERCA. However, we observed that binding of Mg^2+^ and K^+^ induce different structural dynamics of the cytosolic headpiece of SERCA. Structural comparison between the crystal structure (**Figure 7A**) and the MD trajectory (**Figure 7B**) of E1•Mg^2+^ showed that Mg^2+^ binding stabilizes a semi-open headpiece conformation of E1 under physiological conditions. Because our simulations were performed in the absence of TNP-AMP, our results indicate that Mg^2+^, and not TNP-AMP, traps SERCA in a semi-open headpiece conformation. This observation is in agreement with crystallographic studies showing that TNP-AMP crosslinks the N domain and P domain [50], but it only produces a slight difference in the orientation of the N domain and a negligible changes (RMSD <0.05 nm) in the global structure of E1•Mg^2+^ [15]. We also found that the A domain undergoes ∼25° counter clock-wise axial rotation about the lipid bilayer normal (**Figure 7B**). This rotation of the A domain in E1•Mg^2+^ destabilizes N-A and A-P interfaces, therefore preventing the formation of a compact cytosolic headpiece of E1•Mg^2+^. In E1•2K^+^, the N domain swings away from A and P domains (**Figure 7C**), indicating that, compared to E1•Mg^2+^, E1•2K^+^ populates a much more open headpiece conformation of SERCA.

Given the intrinsic flexibility of the cytosolic headpiece in solution [13], [51], [52], analysis of RMSD and representative snapshots extracted from the trajectories is not sufficient simultaneously determine the spatial arrangement and the structural dynamics of the cytosolic headpiece. Therefore, we plotted the interdomain distance distributions of E1•Mg^2+^ and E1•2K^+^ to analyze the structural dynamics of the cytosolic headpiece more quantitatively. Interdomain distance distributions of N-A, N-P and A-P domains were calculated using Cα-Cα distances of the following amino acid pairs: K515-T171 (N-A domains); R489-E680 (N-P domains); and T171-E680 (A-P domains). We tested two models for the distance distribution, ρ(*R*), of each Cα-Cα distance pair: a single Gaussian distribution and two Gaussian distributions. All distances calculated from E1•Mg^2+^ and E1•2K^+^ fit very well to an either one or two Gaussian distribution, with correlation coefficient values ≥0.97 and ≥0.99 for a one and two Gaussian distribution, respectively.

**Figure 8:**
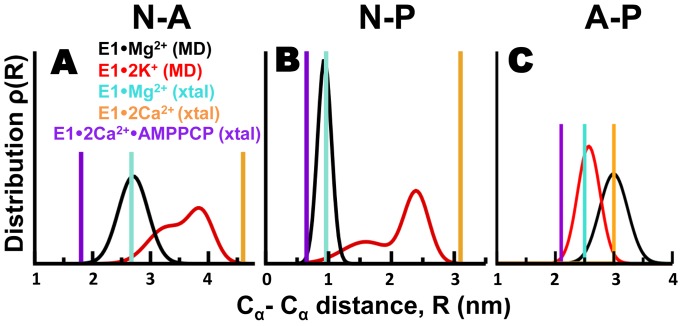
Distance distributions between N, A, and P domains. MD trajectories of E1·Mg^2+^ (black) and E1·2K^+^ (red) were used to calculate Cα-Cα distance distribution between residues (A) K515 and T171 in N and A domains, (B) residues R489 and E680 in N and P domains, and (C) residues T171 and E680 in A and P domains. For comparison, discrete distances for the same pairs of residues were calculated from crystal structures of E1·Mg^2+^ (3w5b in cyan), E1·2Ca^2+^ (1su4 in orange), and E1·2Ca^2+^·AMPPCP (1vfp in purple).

We found that interdomain distances K515-T171 (N-A domains) and R489-E680 (N-P domains) in the trajectory of E1•Mg^2+^ fit to a single Gaussian distribution with means 2.7 and 0.95 nm, respectively (**Figure 8A and B** **, black line**). These values are nearly identical to those calculated in the crystal structure, indicating that the spatial arrangement of N-A and N-P interfaces in the crystal structure of E1•Mg^2+^ is similar to the average geometry observed in solution. Distance between residues T171-E680 (A-P domains) of E1•Mg^2+^ also fits well to a single Gaussian distribution; however, the mean value of the distribution is 0.5 nm larger than that calculated from the crystal structure (**Figure 8**, **black line**). Distances K515-T171 (N-A domains) and R489-E680 (N-P domains) calculated from the trajectory of E1•Mg^2+^ fit to a two Gaussian distribution (**Figure 8A and B** **, red line**). The centers of the bimodal distribution between residues T171-K515 are located at *R* = 3.3 nm and *R* = 3.8 nm, whereas the centers of the distance distribution between R489-E680 are found at *R* = 1.6 nm and *R* = 2.4 nm. These mean distances are substantially larger compared to the distances calculated from the crystal structure, indicating that K^+^ binding to E1 induces an increase in the spatial separation between N-P and N-A domains. The distance distribution plot of the interdomain distance between T171-E680 (A-P) of E1•Mg^2+^ fits a single Gaussian with a mean distance of 2.6 nm, a value very similar to the distance calculated directly from the crystal structure of E1•Mg^2+^ (**Figure 8C** **, red line**).

**Figure 9:**
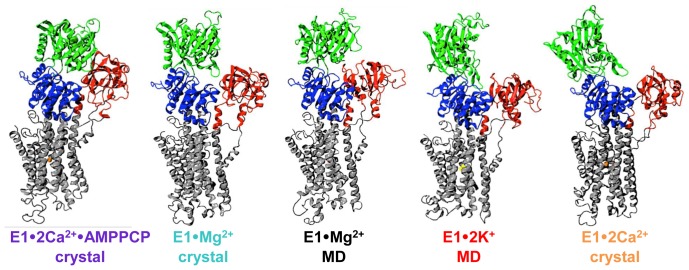
Structural representation of the E1 states of SERCA. We propose that E1 SERCA exists in solution as a broad ensemble of structural states exchanging between open and closed conformations on the µs time scale, and that cation occupancy at the TM transport sites controls this dynamic equilibrium of structural ensembles. Hence, SERCA populates a number of discrete structural states that fall within two opposite ends of the headpiece conformational spectrum: nucleotide-free E1·2Ca^2+^, which features a completely open and mobile headpiece (far right), and nucleotide-bound E1·2Ca^2+^·AMPPCP, which features a compact and relatively rigid headpiece (far left). MD simulation predicts low nucleotide site accessibility for E1·Mg^2+^ and high nucleotide site accessibility for E1·2K^+^.

To broaden the perspective of our analysis, we calculated the distances of residues K515-T171, R489-E680 and T171-E680 in the crystal structures of E1•2Ca^2+^ in the absence and presence of AMPPCP. These structures are relevant to our study because they represent two opposite ends of the headpiece conformational spectrum: the crystal structure of nucleotide-free E1•2Ca^2+^ features a completely open and mobile headpiece conformation [40], whereas the E1•2Ca^2+^•AMPPCP populates a compact and relatively rigid headpiece [53]. Inclusion of these distances in our analysis revealed that that distance distributions from MD simulations fall within the boundaries set by the crystal structures of E1•2Ca^2+^ E1•2Ca^2+^•AMPPCP (**Figure 8A–C** **, orange and purple lines**). Therefore, the E1 SERCA can be described as a broad ensemble of structural states exchanging between open and closed conformations in the µs time scale (**Figure 9**). Despite the differences in time scales used (microsecond vs. millisecond), our simulations agree with recent single-molecule FRET experiments showing that E1 populates several discrete structural states in live cells [51].

## Discussion

### E1 is critical for Ca^2+^-selectivity of SERCA

One of the most interesting aspects of P-type ATPases is their ability to couple ATPase activity with selective metal ion transport. For instance, selective Na^+^ binding to the Na^+^,K^+^-ATPase results from the steric constraints which excludes ions that do not fit metal ion-binding sites [54], [55], [56], [57] Unlike the Na^+^,K^+^-ATPase, the Ca^2+^-binding sites of SERCA can bind metal ions other than Ca^2+^, such as Na^+^ [13], Mg^2+^ [15], [16], and K^+^ (this study). How does SERCA selectively transport Ca^2+^ against other ions in a physiological environment? We found that Mg^2+^ or K^+^ stabilize E1, but fail to induce the structural arrangement of the headpiece necessary for productive ATP hydrolysis. Moreover, interdomain distance distributions revealed important structural differences between E1•Mg^2+^ and E1•2K^+^: Mg^2+^ prevents complete headpiece closure by increasing the distance between P and A domains (**Figure 8C**), whereas K^+^ modulates the N-P interdomain dynamics (**Figure 8B**), inducing a complete opening of the cytosolic headpiece (**Figure 7C**). In line with these observations, previous MD simulations of apo E1 starting from an open headpiece conformation showed that Na^+^ binding to the Ca^2+^-binding sites induces a closure of the headpiece but without the correct alignment of the nucleotide-binding and phosphorylation sites necessary for phosphate transfer [13]. These findings confirm previous observations suggesting that the allosteric signal induced by different metal ions regulate the structural dynamics of the cytosolic headpiece in solution [12], [13]. We propose that the ability of E1 to populate different arrangements of the cytosolic headpiece in the presence of a variety of bound metal ions constitutes a checkpoint following E2-to-E1 transition to couple ATP hydrolysis exclusively with Ca^2+^ binding.

Although E309 spends substantially more time facing the lumen than the cytosol, SERCA is unable to form site II under physiological conditions. The inability of E1•Mg^2+^ and E1•2K^+^ to occlude metal ions in the site II has an important functional consequence: the lack of negative charge neutralization around E309 prevents SERCA from adopting a catalytically competent conformation. The importance of charge neutralization of site II for ATPase activity was experimentally demonstrated in a recent study by Claussen et al., who solved the crystal structure of SERCA mutant E309Q in the presence of Ca^2+^ and AMPPCP [58]. The structure of E309Q mutant revealed the presence of two Ca^2+^occupying sites I and II; however, this E1•2Ca^2+^structure features a headpiece conformation that is not suitable for ATP hydrolysis. Kinetic experiments further showed that E309Q SERCA hydrolyzes ATP, but at a very low maximum rate; the negative effect on ATPase activity was attributed to the lack of charge neutralization around E309, which prevents the A domain from adopting the correct position required for phosphorylation [58]. Therefore, the inability of E1•Mg^2+^ and E1•2K^+^ to neutralize the negative charge around E309 constitutes another crucial checkpoint necessary to prevent unproductive ATP hydrolysis in the absence of bound Ca^2+^.

### E1•Mg^2+^ is an inhibited state of SERCA

Based on crystallographic data, two hypotheses on the physiological relevance of E1•Mg^2+^ were proposed by two groups: on one hand, Toyoshima et al. proposed that E1•Mg^2+^ is an obligate intermediate in the E2-to-E1•2Ca^2+^ transition of SERCA [15]. Toyoshima et al. also suggested that Mg^2+^ binds weakly to the E1, and facilitates the formation E1•2Ca^2+^ through a mechanism involving Mg^2+^-Ca^2+^ exchange (**Figure. 1**)[15]. On the other hand, Winther et al. proposed an opposite hypothesis, in which Mg^2+^ binding slows down Ca^2+^ binding, therefore having an inhibitory effect on SERCA [16]. MD simulation of E1•Mg^2+^ showed that Mg^2+^ binds tightly and with limited mobility to site I′. Furthermore, under physiological conditions, E1•Mg^2+^ did not exchange metal ions in the site I′ in the microsecond time scale. These observations suggest that metal ion exchange at site I′ in E1•Mg^2+^ occurs in much longer time scales, which might result slow Mg^2+—^Ca^2+^ exchange rates. The differences in binding energy found between Ca^2+^ and Mg^2+^ also excludes the Mg^2+^-Ca^2+^ exchange proposed in the model of the catalytic cycle of SERCA [15], as this exchange probably has a high-energy barrier under physiological conditions. These observations suggest that Mg^2+^ binding to site I′ has an inhibitory effect on SERCA. In addition, we found that unlike E1•2K^+^, the cytosolic headpiece of E1•Mg^2+^ is not mobile in solution. In particular, distance distributions between residues R489 and E680 features a narrow peak width with a mean of 0.95 nm, a value that is only ∼0.3 nm different from that calculated in the crystal structure of E1•Ca^2+^•AMPPCP (**Figure 8B**). These observations indicate that N-P interface of E1•Mg^2+^ is structurally restrained in the microsecond time scale, which could hinder nucleotide binding/exchange. These observations suggest that E1•Mg^2+^ represents an inhibited state of the pump. This finding is supported by previous experimental studies. For instance, transient kinetic experiments showed that Mg^2+^ competitively inhibits SERCA by forming a dead-end complex, blocking the ability of Ca^2+^ to reverse the catalytic cycle to form ADP-sensitive, from ADP-insensitive, phosphoenzyme [21]. More recently, radioisotopic and colorimetric assays were used to simultaneously quantify radioactive ^45^Ca^2+^ accumulation in microsomes and ATPase activity of SERCA. These experiments revealed that Mg^2+^ concentrations higher than 5 mM competitively inhibited Ca^2+^ binding sites [59].

Fluorescence experiments showed that in the absence of Ca^2+^, Mg^2+^ induces a pH-dependent change in SERCA fluorescence. In these assays, a minimal change in fluorescence was observed at acidic pH [10]. Based on these observations, the change in fluorescence at neutral or alkaline pH was attributed to the possibility of Mg^2+^ competing with Ca^2+^ for binding to one of the Ca^2+^-binding sites of SERCA [10], [60]. However, tryptophan fluorescence assays of SERCA mutant E309Q excluded this possibility, as binding of Mg^2+^ to the Ca^2+^-deprived E309Q mutant raises fluorescence, whereas binding of Ca^2+^ does not [61]. Based on these fluorescence patterns, it was proposed that in a solution containing 100 mM K^+^, and 5 mM Mg^2+^, it is unlikely that Mg^2+^ binds to the Ca^2+^-binding sites [61]. Our MD simulations agree with these experiments showing that, Mg^2+^ does not reach the Ca^2+^ -binding sites under physiological conditions. Although we do not rule out the possibility that E1•Mg^2+^ exists in solution, it is likely that the fraction of this state is much smaller compared to other metal-bound E1 states, i.e., E1•2K^+^. We also do not rule out the possibility that Mg^2+^ participates at particular steps of the E2-to-E1•2Ca^2+^ transition. For instance, fluorescence spectroscopy experiments have shown that Mg^2+^ plays a role in the Ca^2+^ -binding mechanism; however, these experiments suggested that Mg^2+^ probably binds to a site other than site I′ [7].

### E1•2K^+^ is a functional state of SERCA

Under physiological conditions and in the absence of bound Mg^2+^, two K^+^ ions rapidly occupy to the empty Ca^2+^ sites of E1. We found that K^+^ is capable to fulfill the partial charge neutralization requirements of the Ca^2+^ sites, an essential requirement of the structural stability of the TM domain of SERCA [2]. E1 binds two K^+^ ions in a novel fashion to unique rearrangements of the Ca^2+^-binding sites. The arrangement of the Ca^2+^ sites is in some aspects similar to that induced by Ca^2+^ (**Figure 2C and D**), suggesting that K^+^ is recognized by SERCA as a native ligand. Furthermore, E1•2K^+^ features an open headpiece structure (**Figure 8**), which could facilitate nucleotide binding/exchange. These findings indicate that, under physiological conditions, E1•2K^+^ is not only structurally stable but also the most populated E1 intermediate state preceding Ca^2+^ binding. If E1•2K^+^ is the most populated E1 intermediate state in solution, what functional role does it play in Ca^2+^ transport? Unlike Mg^2+^, K^+^ not only binds weakly to the Ca^2+^ sites of SERCA, but also induces a geometrical arrangement of site I that is similar to that induced by Ca^2+^, i.e., engaging residues E771, T799 and D800 in K^+^-protein electrostatic interactions (**Figure 2C**). However, K^+^ is unable to engage N768 in metal-protein interactions. These findings suggest that E1•2K^+^ plays a central role in the E2-to-E1•2Ca^2+^ transition.

Moutin and Dupont have previously reported experimental evidence that supports the formation of a functional E1•2K^+^ state, a necessary step for Ca^2+^ binding in the catalytic cycle of SERCA. Moutin and Dupont used stopped-flow experiments to determine the effect of K^+^ on the kinetics of Ca^2+^ binding to and dissociation from SERCA. These experiments revealed that at pH 7.2 and in the absence of Mg^2+^, increasing the K^+^ concentration from 0 to 100 mM produces a 4-fold increase of the rate constant of the Ca^2+^-induced fluorescence change and an 8-fold increase of the rate constant of the EGTA-induced fluorescence change [7]. Rapid filtration assays showed that K^+^ binding increases the rate of ^45^Ca^2+^-^4^°Ca^2+^ exchange reaction. In addition, it was found that K^+^ accelerates the isotopic exchange of the slow-exchanging type in the Ca^2+^ sites. These observations indicate that K^+^ ions interact with Ca^2+^-binding sites in order to accelerate Ca^2+^ binding to and migration across sites I and II. We propose that the E2-to-E1•2Ca^2+^ transition consists of two steps: (i) *Formation of the site I*. Structural comparison between E1•Mg^2+^ and E1•2K^+^ showed that only K^+^ binding induces the formation of a Ca^2+^-bound-like site I (**Figure 2**). Therefore, K^+^ binding to the Ca^2+^ sites is a step necessary to produce a competent site I that is capable of recognizing and binding Ca^2^. (ii) *K^+^-Ca^2+^ exchange*. Following the formation of site I, N768 swings away from site I (**Figure 2**), opening a pathway between site I and the cytosol, facilitating metal ion exchange. K^+^-Ca^2+^ exchange will also be facilitated by weak K^+^-SERCA interactions at sites I and I′.

## Conclusion

Microsecond MD simulations predict that both E1**•**Mg^2+^ and E**•**2K^+^ intermediate states of SERCA exist in solution in the absence of Ca^2+^, with the 2K^+^-bound state being more populated at physiological ion concentrations. Comparison between our MD simulations and published experimental data indicate that E1•Mg^2+^ represents an inhibited state of the pump, whereas E1•2K^+^ is a functional intermediate that plays a central role in the E2-to-E1•2Ca^2+^ transition. E1**•**Mg^2+^ and E**•**2K^+^ are structurally stable but fail to induce the structural arrangement of the headpiece necessary for productive ATP hydrolysis. E1•Mg^2+^ modulates the dynamics of A-P domains, whereas E1•2K^+^ populates an open headpiece structure by increasing the distance between N and P domains. The ability of E1 to populate different arrangements of the cytosolic headpiece in the presence of a variety of bound metal ions constitutes a checkpoint following the E2-to-E1 transition. In addition, E1**•**Mg^2+^ and E**•**2K^+^ are unable to form site II under physiological conditions. The inability of E1•Mg^2+^ and E1•2K^+^ to occlude metal ions results in the lack of charge neutralization around E309. The inability of E1•Mg^2+^ and E1•2K^+^ to neutralize the charge around E309 constitutes another checkpoint necessary to prevent unproductive ATP hydrolysis in the absence of bound Ca^2+^. The structural adaptability and the inability to stabilize site II effectively connects E1 dynamics with Ca^2+^-selectivity. We propose that E1**•**2K^+^ acts as a functional intermediate that accelerates the E2 to E1**•**2Ca^2+^ transition through two mechanisms: by pre-organizing transport sites for Ca^2+^ binding and by facilitating partial headpiece opening prior to Ca^2+^-activation of nucleotide binding. We propose that E1**•**2K^+^ is competent to act as a functional intermediate that regulates the E2 to E1**•**2Ca^2+^ transition, and that both E1**•**Mg^2+^ and E**•**2K^+^ constitute essential checkpoints for selective coupling of Ca^2+^ binding to ATP hydrolysis in the catalytic cycle of SERCA.
